# Database proton NMR chemical shifts for RNA signal assignment and validation

**DOI:** 10.1007/s10858-012-9683-9

**Published:** 2012-11-23

**Authors:** Shawn Barton, Xiao Heng, Bruce A. Johnson, Michael F. Summers

**Affiliations:** 1 Howard Hughes Medical Institute, University of Maryland, Baltimore County, 1000 Hilltop Circle, Baltimore, MD 21250 USA; 2 Department of Chemistry and Biochemistry, University of Maryland, Baltimore County, 1000 Hilltop Circle, Baltimore, MD 21250 USA; 3 One Moon Scientific, Inc., 839 Grant Ave., Westfield, NJ 07090 USA

**Keywords:** RNA, Chemical shift, A-form helices, NMR signal assignment and validation

## Abstract

The Biological Magnetic Resonance Data Bank contains NMR chemical shift depositions for 132 RNAs and RNA-containing complexes. We have analyzed the ^1^H NMR chemical shifts reported for non-exchangeable protons of residues that reside within A-form helical regions of these RNAs. The analysis focused on the central base pair within a stretch of three adjacent base pairs (BP triplets), and included both Watson–Crick (WC; G:C, A:U) and G:U wobble pairs. Chemical shift values were included for all 4^3^ possible WC-BP triplets, as well as 137 additional triplets that contain one or more G:U wobbles. Sequence-dependent chemical shift correlations were identified, including correlations involving terminating base pairs within the triplets and canonical and non-canonical structures adjacent to the BP triplets (i.e. bulges, loops, WC and non-WC BPs), despite the fact that the NMR data were obtained under different conditions of pH, buffer, ionic strength, and temperature. A computer program (RNAShifts) was developed that enables convenient comparison of RNA ^1^H NMR assignments with database predictions, which should facilitate future signal assignment/validation efforts and enable rapid identification of non-canonical RNA structures and RNA-ligand/protein interaction sites.

**Electronic supplementary material:**

The online version of this article (doi:10.1007/s10858-012-9683-9) contains supplementary material, which is available to authorized users.

## Introduction

RNA molecules participate in a large and expanding array of known biological functions including gene regulation, maintenance of sub-cellular and viral structure, intracellular trafficking, antiviral restriction, catalysis, and, of course, propagation of genetic information (Korostelev and Noller 2007; Steitz 2008; Bessonov et al. 2008; Boisvert et al. 2007; Wakeman et al. 2007; Edwards et al. 2007; Bartel 2004; Kim 2005; Hassouna et al. 1984; Brodersen and Voinnet 2006; Doudna and Rath 2002; Ponting et al. 2009). Like proteins, the functional activities of most RNAs are intrinsically linked to their structures. Unfortunately, although a wealth of structural information is currently available for functionally active proteins and protein domains, structural information for functionally relevant RNAs remains relatively limited. Thus, the Protein Data Bank (PDB; http://www.rcsb.org/pdb/home/home.do) currently contains more than 55,000 protein structure depositions, whereas the Nucleic Acid Database (NDB; http://ndbserver.rutgers.edu/) contains atomic coordinate depositions for fewer than ~2,100 RNAs and protein/ligand-RNA complexes, of which ~1,600 were determined by X-ray crystallography and ~500 by NMR spectroscopy. Conformational heterogeneity and the presence of a relatively uniform, negative surface charge can hinder structural studies by X-ray crystallography, and as discussed below, difficulties associated primarily with limited chemical shift dispersion have generally limited NMR applications to relatively small RNAs. For these reasons, much of what is known about the structures of biologically functional RNAs (primarily secondary structure information) has been obtained by chemical and enzymatic accessibility mapping experiments, coupled with phylogenetic and free energy calculations. Although RNA probing methodologies are potentially very powerful and have been widely applied (Peattie and Gilbert 1980; Ehresmann et al. 1987; Stern et al. 1988; Forconi and Herschlag 2009; Weeks 2010), interpretation of the data can be problematic, particularly for RNAs that exist as equilibrium mixtures of multiple conformational species (see for example, Kladwang et al. 2011; Houck-Loomis et al. 2011; Lu et al. 2011a,b; Miyazaki et al. 2010).

NMR is a potentially powerful tool for probing RNA structure (Wüthrich 1986; Allain and Varani 1997; Lukavsky and Puglisi 2005), but its application to larger RNAs can be complicated by a number of factors. Inter-residue scalar couplings are generally weak, limiting the utility of “through bond” inter-residue connectivity experiments for signal assignment. The most commonly used assignment approach involves identification of sequential inter-residue NOE connectivities (Wüthrich 1986), but even this approach can be problematic for modest sized RNAs (ca. 25–60 nucleotides). Although resolution can be increased by ^1^H–^13^C heteronuclear spectral editing (Peterson et al. 2004; D’Souza et al. 2004; D’Souza and Summers 2004; Davis et al. 2005; Batey et al. 1995; Batey et al. 1992; Nikonowicz and Pardi 1992; Nikonowicz et al. 1992; Michnicka et al. 1993; Kim et al. 1995; Xu et al. 1996; Kim et al. 2002; Lukavsky et al. 2003; Lu et al. 2009), chemical shift dispersion is relatively limited (Allain and Varani 1997; Lukavsky and Puglisi 2005), and severe dipolar broadening of the aromatic ^1^H–^13^C signals that are critical for structural analysis can preclude detection of ^1^H–^13^C correlation NMR signals in larger RNAs (Lu et al. 2011a). In addition, interproton distances between elements of secondary structure in larger RNAs typically exceed those required for NOE detection (Lu et al. 2009; Tolbert et al. 2010). Thus, high-resolution NMR-based structural studies have been applied mainly to relatively small RNAs: Of the 496 RNA NMR structures that have been deposited in the NDB, only 19 contain 60 or more nucleotides; the largest is a symmetrical dimer of 132 nucleotides (two 66 nucleotide subunits), and the average size is ~27 nucleotides.

One approach for addressing issues of signal degeneracy involves the application of traditional 2D NOESY experiments to RNA samples that are site- and/or nucleotide-specifically labeled with deuterium (Miyazaki et al. 2010; D’Souza et al. 2004; Davis et al. 2005; Kim et al. 1995; Lu et al. 2009; Zhou et al. 2006; Nelissen et al. 2008; Heng et al. 2012; Duss et al. 2012). ^2^H-isotope edited 2D NMR has enabled nearly complete assignment of the aromatic, H_1_′, H_2_′, and H_3_′ ribose signals of RNAs containing up to 132 nucleotides (Miyazaki et al. 2010), and has also enabled assignment of selected residues within a 720 nucleotide RNA (Lu et al. 2011a; Heng et al. 2012). This approach, which involves comparison of high resolution 2D NOESY spectra obtained for multiple, differentially ^2^H-labeled samples, avoids relaxation problems associated with aromatic ^1^H–^13^C spectral editing and enables observation of signals in 2D ^1^H–^1^H NOESY spectra for protons with T_2_ values as short as 8 ms (Lu et al. 2011a). Although resolution and sensitivity can be improved dramatically by nucleotide-specific deuteration, signal overlap can still hinder the assignment process for RNAs comprising more than 150 nucleotides (Summers and coworkers, unpublished).

NMR chemical shifts have been widely utilized for NMR signal assignment and structural studies of proteins (for examples see: Grzesiek and Bax 1993; Wishart and Sykes 1994; Wishart et al. 1991, 1992; Cavalli et al. 2007; Shen et al. 2008; Wishart et al. 2008). Although relationships between ^13^C chemical shifts and RNA structure have been identified (Ebrahimi et al. 2001; Fares et al. 2007; Ohlenschlager et al. 2008), and ^15^N NMR chemical shifts have been incorporated into a probabilistic approach for automated assignment of RNA imino groups (Bahrami et al. 2012), heteronuclear NMR chemical shifts have not been widely exploited for RNA studies (Lam and Chi 2010; Aeschbacher et al. 2012). On the other hand, Wijmenga and co-workers showed that non-exchangeable ^1^H NMR chemical shifts for A-form helical residues could be back-calculated from a given 3D RNA structure (Cromsigt et al. 2001). For 28 examples tested, the back-calculated shifts were in good agreement with shifts reported in the Biological Magnetic Resonance Bank (BMRB; www.bmrb.wisc.edu), and some general ^1^H NMR chemical shift trends were identified (Cromsigt et al. 2001). Here we report a detailed analysis of the H_8_, H_2_, H_6_, H_5_, H_1_′, H_2_′, and H_3_′ proton NMR chemical shifts that have been deposited in the BMRB. After correcting for differences in chemical shift referencing and sample conditions, excellent correlations were observed, despite the fact that the data were obtained over a wide range of sample conditions. Our findings confirm and quantify previously identified trends and identify new sequence- and structure-dependent chemical shift correlations that can be used for assignment and/or validation of non-exchangeable ^1^H NMR chemical shifts and for the identification of non-canonical RNA structural features and intermolecular interaction sites.

## Methods

NMR data were analyzed using “RNAShifts”, a program designed to download and analyze RNA ^1^H NMR chemical shifts that have been deposited in the BMRB. (Locally derived shifts that have yet to be deposited can also be analyzed). All 131 depositions available in the BMRB were used in the current analysis except BMRB ID 5170, 6814, 4816, 15697, 15915, 5023, 4253, 4894, and 15257, which could not be reliably used because either the BMRB assignments didn’t match the published PDB assignments, or because there was no associated publication or PDB file that could be used to identify RNA secondary structure. As additional input, files were manually generated for each deposition, based on published structural studies, that identify for each residue (1) whether or not the residue is base-paired, (2) the nature of the base-pairing partner, (3) any long-range intra- and/or inter-molecular interactions (e.g., sites of protein binding or participation in A-minor or other RNA–RNA contacts), (4) participation in structured (e.g., GNRA; G/g = guanosine, N/n = any nucleotide; R/r = purine; A/a = adenosine) or unstructured loops. A representative input file is shown in Supplementary Table S1.

**Fig. 1:**
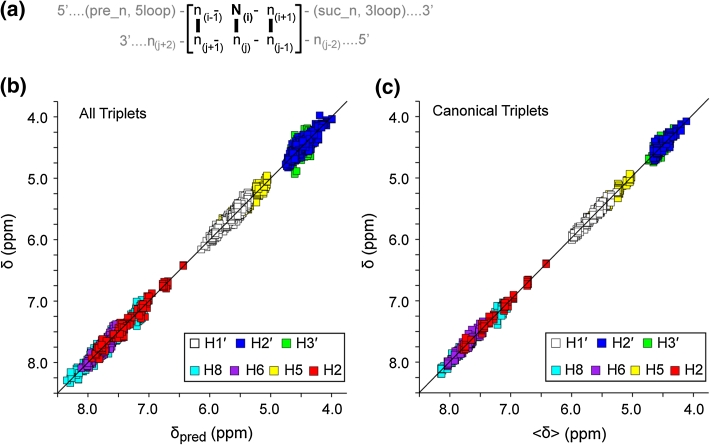
**a** Definitions used for base pair triplets. The chemical shifts of the N_(i)_ residue are analyzed in this work, and this strand may be preceded by a base-paired (WC or GU wobble) nucleotide (pre_n) or a non-base paired residue (5loop), or followed by a base-paired residue (suc_n) or non-base paired residue (3loop). **b** Plot of the database chemical shift (automatically re-referenced as described in the text) (δ) versus calculated chemical shift (δ_pred_) for the 3758 assignment depositions utilized in the present study (rms deviation = 0.056). **c** Plot of δ versus mean chemical shift (〈δ〉) for residues in canonical triplets (triplets that contain only GC and/or AU base pairs and are both preceded and followed by a GC and/or AU base pair) (rms deviation = 0.043)

The analysis focused on shifts reported for the non-exchangeable H_8_, H_2_, H_6_, H_5_, H_1_′, H_2_′ and H_3_′ protons of the central base pair of three consecutive canonical Watson–Crick base-pairs (WC-BPs) (here called WC-BP triplets: ([5′−n_(i−1)_−N_i_−n_(i+1)_]:[5′−n_(j−1)_−n_j_−n_(j+1)_]; N_i_ = nucleotide for which the NMR shifts are being evaluated; n = neighboring nucleotides), Fig. 1a. As additional parameters, we denoted if the n_(i−1)_:n_(j+1)_ or n_(i+1)_:n_(j−1)_ base pairs were at terminating positions in the RNA, and we identified the secondary structural elements adjacent to the WC-BP triplets (canonical or non-canonical WC-BP, bulges, loops, long-range RNA–RNA interactions, and RNA–protein/ligand interactions), Table S1.

We chose a relatively conservative approach in modeling the effect of the neighborhood of each central base pair. This was done because there are still, especially in comparison to proteins, relatively few chemical shift assignment sets for RNA deposited at the BMRB. Rather than using any non-linear or neural network approach we used an approach similar to the chemical shift increment method of Pretsch as used in predicting spectra of small organic molecules (Pretsch et al. 2009). Thus, for the central residue of each WC-BP triplet, we defined the attributes describing the neighborhood of the central nucleotide as described above, and calculated the contribution that each attribute makes to the predicted chemical shift. The predicted chemical shift is then a base chemical shift plus the linear contribution of the value corresponding to each attribute present in that nucleotide’s environment. The contribution of each attribute was calculated by linear regression of the chemical shifts in our database of RNA chemical shifts with the set of explanatory variables represented by the neighborhood attributes. The constant term of our regression model corresponds to a nucleotide embedded in a triplet of Watson–Crick base pairs with a U (uridine) flanking it on both the 5′ and 3′ sides and Watson–Crick base-paired nucleotides at the 5′ and 3′ ends of the triplet.

Our analysis included a total of 15 potential variables, Table 1, of which only some might potentially contribute significantly to the shift of a specific atom in a given central nucleotide. Because the approach includes a large number of independent variables relative to the chemical shift datasets, there was a significant danger of over-fitting using a conventional linear regression algorithm. Over-fitting can lead to excellent prediction of the training set, but poor predictive capability on novel datasets. To minimize the risk of over-fitting we chose an algorithm, Pace Regression (Projection Adjustment by Contribution Estimation), that is capable of assessing the importance of each of the parameters. Calculations were performed using the Weka Machine Learning and Data Mining Library system, which allowed us to perform a statistical analysis of the prediction model (Witten et al. 2011). Pace Regression is a linear regression system that uses various information criteria to assess the degree of importance of the regression variables (Wang and Witten 2002). Thus it provides one solution to the subset selection problem: which subset of a set of potential regressors is the appropriate set to explain the data, and thereby minimize the risk of overfitting and maximize the predictive capability on previously unseen data.

**Table 1:** Sequence variables and chemical shift corrections calculated by Pace regression

Atom	const	pre_a	pre_c	pre_g	pre_gu	pre_ug	suc_a	suc_c	suc_g	suc_gu
Part 1										
A–H_2_	7.0299	0.6672	0.2521	0.6899	0.7637	0.2555	−0.5934	0.0658	0.2814	−0.3321
A–H_8_	8.1525	−0.4169	−0.1145	−0.3709	−0.3808	0.2107	−0.1006	0.0379	−0.0723	0.0458
G–H_8_	7.7036	−0.5622	−0.1595	−0.497	−0.5083	0.2215	−0.0383	0.0562	−0.0265	0.0369
C–H_5_	5.6724	−0.4611	−0.1625	−0.4304	−0.2815	0.0377	−0.0222	−0.0368	−0.0249	−0.032
U–H_5_	5.5639	−0.5133	−0.1778	−0.5018	−0.3641	−0.0469	0.0333	0.0165	0	0.0348
C–H_6_	7.8627	−0.3901	−0.0867	−0.2215	−0.2059	0.085	−0.0253	0.0363	−0.0288	−0.0833
U–H_6_	7.9946	−0.3643	−0.1083	−0.2523	−0.3392	0.0832	−0.0402	0.0332	−0.0555	−0.0809
A–H_1_′	5.9905	−0.1042	−0.05	0	−0.0639	0	−0.0823	0	−0.0324	0.0528
G–H_1_′	5.7539	−0.1822	−0.0584	−0.019	−0.068	−0.0373	0	0.0216	0.0198	0.0777
C–H_1_′	5.56	−0.1805	−0.0825	−0.0446	−0.0492	0.039	−0.0226	−0.0139	0.0134	0.0711
U–H_1_′	5.6203	−0.1679	−0.0865	−0.1049	−0.1992	0.0234	−0.0405	0.0097	0.0196	0.0571
A–H_2_′	4.4575	−0.0269	0.0268	0.0309	−0.0499	0.1174	0.1041	0.0496	0.1613	0.1416
G–H_2_′	4.4582	−0.0666	0.0389	0	0	0.0794	0.1367	0.0248	0.1286	0.0705
C–H_2_′	4.3454	−0.1298	0.0188	0.0499	−0.0867	0.0363	0.1475	0	0.1557	0.1724
U–H_2_′	4.4078	−0.0599	0.0404	0.0658	−0.0445	0.0618	0.0807	0.059	0.174	0.1431
A–H_3_′	4.6788	−0.1275	−0.0229	−0.0794	−0.083	0.0436	−0.0377	0.006	0.0105	0.0593
G–H_3_′	4.5076	−0.1292	−0.029	−0.0537	−0.1527	0.0644	0.0445	0.0459	0.0333	0.0674
C–H_3_′	4.4794	−0.1336	−0.0189	−0.0431	0.0221	0.0364	0.0498	−0.0147	0.0454	−0.043
U–H_3_′	4.495	−0.119	−0.0065	−0.0447	0.0458	0.0506	0.1024	0.0229	0.0657	0.0839

Atom	suc_ug	5ter	3ter	3loop	5loop	GU	corr	rms	nobs	xcorr	xrms	ntrim
Part 2												
A–H_2_	0.0804	0.1558	0	0.0274	0.0622	0	0.9868	0.0562	162	0.9845	0.0608	1
A–H_8_	−0.0261	0.3206	0	−0.0175	0.08	0	0.9535	0.0575	157	0.9406	0.065	0
G–H_8_	−0.0671	0.3563	−0.0607	−0.0253	0.0332	−0.0529	0.9655	0.0658	288	0.9612	0.0697	2
C–H_5_	−0.0958	0.1245	−0.016	−0.0205	0.01	0	0.9674	0.0478	256	0.9635	0.0505	5
U–H_5_	0.047	0	0	0	0.0216	0.2747	0.9692	0.0537	178	0.9623	0.0594	0
C–H_6_	−0.1132	0.2326	−0.0359	−0.0428	0.0311	0	0.9332	0.0558	260	0.9249	0.0591	0
U–H_6_	−0.0457	0	−0.0287	−0.0341	0.0268	−0.0412	0.9444	0.0446	172	0.9331	0.0489	4
A–H_1_′	−0.0353	0.0966	0	−0.0173	0.0061	0	0.8476	0.0332	157	0.7905	0.0384	0
G–H_1_′	0	0.1435	−0.0376	0	0	0	0.9278	0.0415	284	0.9209	0.0434	3
C–H_1_′	−0.0224	0.1351	0.0114	−0.0168	0.0121	0	0.8355	0.0427	253	0.7872	0.0482	5
U–H_1_′	0.0328	0	0.0183	0.0145	0.0403	−0.0316	0.7411	0.053	172	0.6451	0.0609	1
A–H_2_′	0.1044	0.0702	0	−0.0115	0.0291	0	0.7512	0.0618	143	0.6531	0.0714	0
G–H_2_′	0.1022	0.074	−0.1122	−0.0326	−0.0257	0.1211	0.8675	0.0516	246	0.8359	0.057	2
C–H_2_′	0.1198	0.085	−0.1316	0	0	0	0.9014	0.0589	217	0.8774	0.0654	4
U–H_2_′	0.1691	0	−0.229	−0.0488	−0.03	−0.3095	0.92	0.0609	146	0.8999	0.0679	2
A–H_3_′	0.0283	0.0235	0	−0.0248	0.0202	0	0.5997	0.064	129	0.4933	0.0708	0
G–H_3_′	−0.0887	0.1061	−0.0416	−0.0146	0.0244	−0.1527	0.7307	0.0807	226	0.6391	0.0914	3
C–H_3_′	−0.1182	0.1326	−0.0178	0.0096	−0.0224	0	0.7442	0.062	191	0.6942	0.0669	4
U–H_3_′	−0.0268	0	−0.0432	−0.0187	0.0159	0.0266	0.7047	0.0482	121	0.5928	0.0552	2

Output from the Pace Regression analysis. Each row represents an individual atom type in the specified nucleotide (e.g., A-H_2_ is the H_2_ proton of Adenine). The column labeled *const* represents the chemical shift of that atom in the triplet uXu when none of the additional attributes represented in subsequent columns are present. Contributions with values equal to 0 represent attributes that the Pace Regression algorithm found could not be supported by the data and were thereby automatically excluded from the regression analysis. The contribution from columns labeled pre_x and suc_x, where x is a,c,g, or gu are used where the preceding or succeeding nucleotide is not a u. A GU attribute represents the case where the nucleotide is in a GU, rather than GC, base pair, and can apply to the i − 1 (pre_gu), i + 1 (suc_gu) or central (GU) triplet (with the same approach used for UG wobbles). The 5ter attribute indicates the triplet is at the 5′ end (so there is no i − 2 nucleotide), and 3ter indicates the triplet is at the 3′ end (so there is no i + 2 nucleotide). The loop attributes indicate that the i − 2 (5loop) nucleotide or i + 2 (3loop) nucleotide is in a loop or mismatched base pair. The columns labeled *corr* and *rms* represent the correlation coefficient (corr) and the square root of the mean of squared deviations between predicted and experimental values (rms) for all the data in the fit. The columns labeled *xcorr* and *xrms* represent the same values, but calculated with 10-fold stratified cross-validation. The column labeled *nobs* represents the number of observations available and *ntrim* the number that were automatically eliminated as outliers

Use of Weka provided not only access to Pace Regression, but also various assessments of the quality of the predictions. In particular, we used 10-fold stratified cross-validation during our analysis. Rather than providing correlation coefficients and root mean squared (rms) deviations of the predictions using all the data in the prediction, this technique trains the model on 90 % of the data and then assesses the results of predicting the remaining 10 % of the data. The process is repeated 10 times, using a different subset of the data each time and derives the correlation coefficients and rms deviations based on the whole process. Pace regression was used independently on each atom type present in each of the four central nucleotides for a total of 19 regression calculations.

We were unable in our analysis to adequately identify and control for sample conditions (pH, temperature, ionic strength, etc.) and unusual molecular conformation, and there is a significant possibility of misassignment, especially of some atom types. Therefore, after dropping a single obvious major outlier, we minimized these effects by automatically trimming outliers and automatically adjusting the reference for the chemical shift sets. Automated outlier elimination was performed by running two passes of the Pace Regression for each atom/central nucleotide. In the first pass, the rms deviations between the experimental and predicted values were calculated using all of the data. Any data values that deviated from the predicted values by more than three times the rms deviation value were dropped, and a second pass of the Pace Regression was performed on the now trimmed dataset. Automatic re-referencing was achieved by performing the above analysis (including outlier detection) twice. In the first of these passes, the mean error of prediction was calculated for all the shifts from each BMRB file. Prior to the second pass, each shift was corrected by the mean deviation calculated for the corresponding BMRB file. The chemical shift corrections determined by this approach are listed in Table S3.

The RNAShifts program was written using JTcl (http://jtcl.kenai.com) and Swank (http://swank.kenai.com), which are the Java implementations of the Tcl programming language and Tk graphical user interface toolkit (Ousterhout and Jones 2010). The analysis mode is run in three stages. The first loads BMRB files (fetching them from http://bmrb.wisc.edu if necessary), extracts chemical shifts, and then uses the input template to assign attributes to each shift. The second stage reads the output of the first stage and generates input files in the format used by Weka. The third executes Weka multiple times for each proton type, manages the two passes used for outlier detection and generates various statistical output files. The graphical interface module allows plotting predicted and experimental data subject to various criteria for choosing subsets of the data and attributes for plotting. The RNAShifts program is available upon request from the author (BAJ).

## Results and discussion

### Outlier chemical shifts

The statistical analysis described above identified 65 chemical shift assignments from the full BMRB database that, after automated re-referencing, deviated from expected values by more than 3 standard deviations. Seven of these assignments were associated with earlier publications from the M.F.S. laboratory, and inspection of the original NMR spectra revealed that these signals had been erroneously assigned (corrections to BMRB files 15113 and 17083 have now been made). We also discovered relatively large systematic chemical shift variations for one of our earlier depositions (BMRB ID 6094) that were associated with improper chemical shift referencing (the residual water signal at 35 °C was erroneously assigned a chemical shift of 4.792 ppm). We therefore updated the BMRB with the modified values, which were used in the present analysis. Based on examination of published NMR spectra, we were able to correct 19 additional assignments in the BMRB—in many cases, the signals had been properly assigned in the published spectra but improperly recorded in the BMRB files. In all cases, the re-assigned (or typo-corrected) shifts were well within the 3-standard deviation cutoff. We were unable to determine the nature of the deviations observed for the remaining 38 outliers because relevant regions of the NMR spectra were not provided in the original publications, and these 38 assignments were not used in subsequent analyses. The majority of these outliers were associated with ribose protons, of which 17 were for highly overlapping H_2_′ and H_3_′ proton signals. Thus, of the 3,796 available chemical shifts, 3,758 were retained for analysis and 38 (1 %; mostly ribose assignments) were excluded.

Chemical shifts that were either re-assigned or excluded are summarized in Supplementary Table S2, and referencing corrections employed for all of the utilized depositions are summarized in Supplementary Table S3. The final dataset included values for the central base pairs of all of the 4^3^ possible combinations of WC-BP triplets, with as few as one, and as many as 23, assignments for each of the possible combinations. A total of 137 additional triplets that contain G:U base pairs were also included in the analysis. As shown in Fig. 1b, the retained and re-referenced BMRB shifts (δ) were in good agreement with predicted shifts (δ_pred_) (rms deviation for the entire dataset = 0.056). Good agreement was also obtained when training was performed using a two-fold cross-validation analysis, in which half of the data were used for training and half for validation (rms = 0.069 ppm), and when training was performed with 60 % of randomly-ordered BMRB entries and validation assessed with the remaining 40 % of the data (rms = 0.063, averaged over all atom types).

### Chemical shift trends for canonical triplets

**Fig. 2:**
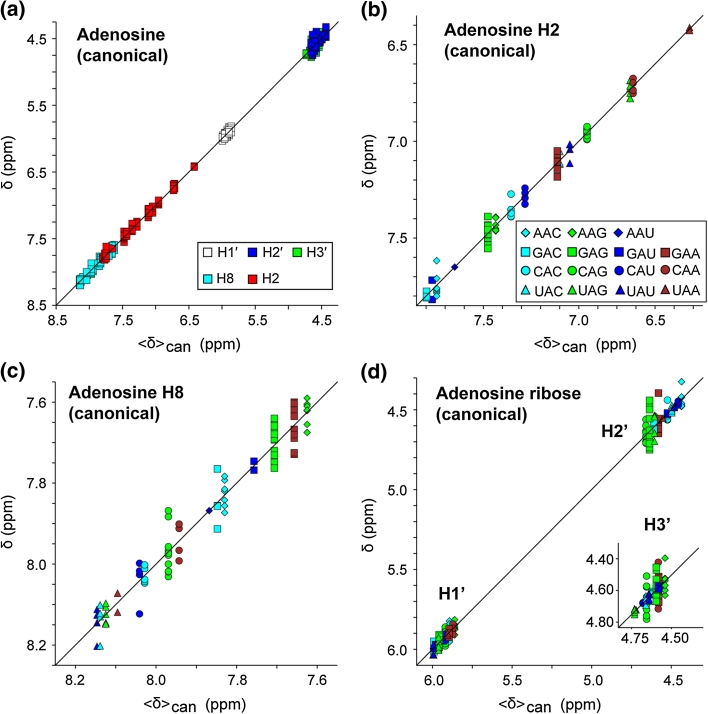
Plots of re-referenced ^1^H NMR chemical shifts (δ) reported for the central adenosine residues within canonical triplets (as defined in text and Fig. 1 caption) versus mean shifts calculated for canonical triplets (〈δ〉_can_). **a** Data are shown for all adenosine protons grouped by atom type (*symbols* defined in **a** inset). **b**–**d** Expansions showing data grouped according to triplet sequence for the adenosine H_2_ (**b**), H_8_ (**c**) and ribose (**d**) protons (*symbols* defined in **b** *inset*)

The re-referenced NMR chemical shifts (δ) were generally in good agreement with the mean shifts calculated for each unique sequence/atom type (〈δ〉). For example, excellent correlations were observed in a plot of δ versus 〈δ〉 for the central residues of “canonical triplets,” defined here as a triplet that contains only GC and/or AU base pairs *and* are both preceded and followed by canonical GC or AU base pairs (rms deviation = 0.043), Fig. 1c. The database utilized does not contain chemical shift values for aAa and uCa canonical triplets, nor for the H_2_′ and/or H_3_′ protons of the following canonical triplets: aAu (H_2_′, H_3_′), uGa (H_2_′, H_3_′), aUu (H_2_′, H_3_′), gGu (H_3_′), aCc (H_3_′). (Note that data were available for non-canonical forms of these triplets and were included in the analysis). There were no significant differences in correlation coefficients obtained upon fitting δ versus 〈δ〉 for the A, G, C and U nucleotides, but as observed in plots of δ versus δ_pred_, greater scatter was generally observed for the H_2_′ and H_3_′ protons, Fig. 2a.

**Fig. 3:**
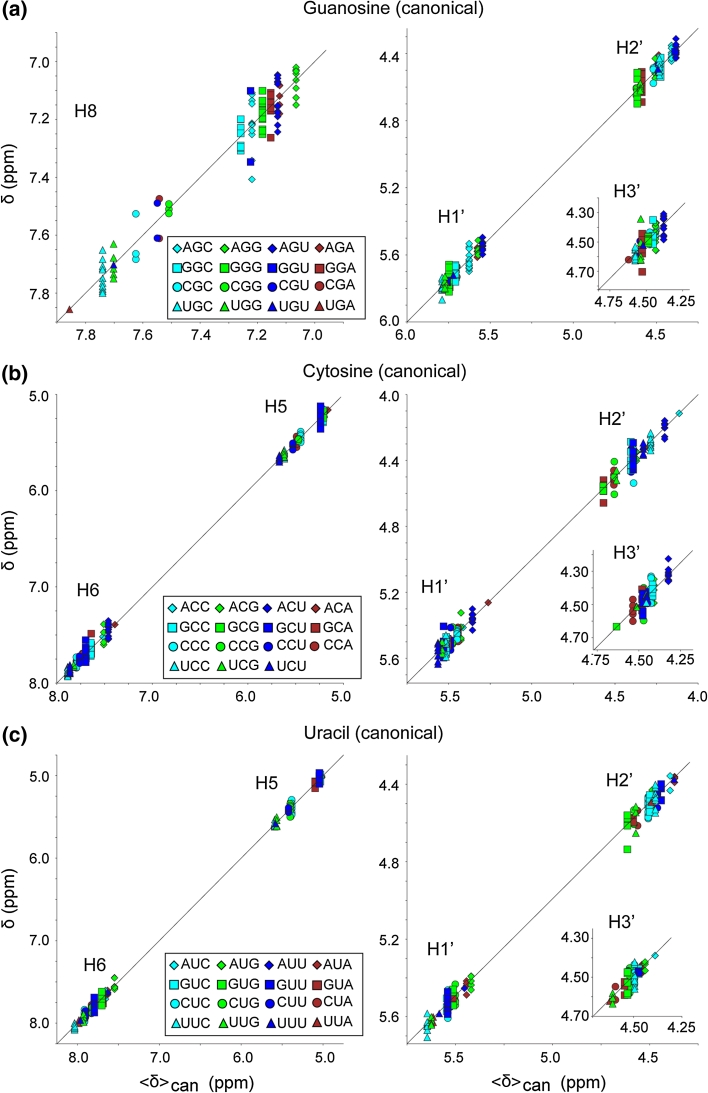
Plots of re-referenced ^1^H NMR chemical shifts (δ) reported for the central guanosine (**a**), cytosine (**b**) and uracil (**c**) residues within canonical triplets (as defined in text and Fig. 1) versus mean shifts calculated for canonical triplets (〈δ〉_can_). Data are grouped by atom type as defined in panel *insets*

**Fig. 4:**
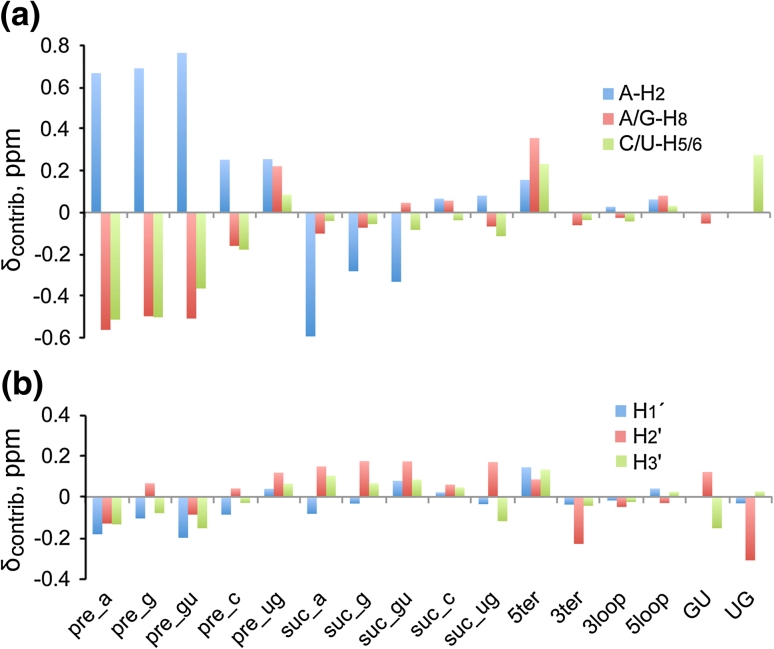
Plot of the chemical shift contributions (δ_contrib_) of each attribute relative to a canonical uNu triplet as obtained via Pace Regression for aromatic (**a**) and ribose (**b**) proton assignments (positive values denote downfield shifts). Data in these plots are derived from Table 1. For simplification, data for aromatic protons with similar trends in their response to the attributes were combined, and within each group of proton type, the largest absolute value is plotted. Because this procedure can mask the details of individual proton types one should use this plot for observing general trends and refer to the specific contributions in Table 1

^1^H NMR chemical shift trends were readily observed in plots that compare δ with the mean shift calculated for canonical triplets (〈δ〉_can_), and with the coefficients obtained with the Pace Regression analysis. Plots of δ versus 〈δ〉_can_ for the n-A-n canonical triplets are shown in Fig. 2, and data for the n-G-n, n-C-n and n-U-n canonical triplets are plotted in Fig. 3. The contributions of the attributes calculated by Pace Regression are plotted in Fig. 4. The observed trends are consistent with several generalized correlations identified by Wijmenga and co-workers (Cromsigt et al. 2001). For example, δ values for purine-H_8_ protons in canonical triplets are highly sensitive to the nature of the 5′-residue within the triplet, with 5′-purines associated with more upfield chemical shifts. We further observe that 5′-uridines induce a larger downfield H_8_ shift than 5′-cytidines (Figs. 2c, 3a), and that the H_8_ chemical shift is also sensitive to the nature of the 3′-residue, Figs. 2c and 3a. For example, the A-H_8_ 〈δ〉_can_ values observed for n-A-a canonical triplets are consistently downfield relative to those observed for n-A-g canonical triplets, Fig. 2c, and a similar trend is observed for n-G-a versus n-G-g triplets, Fig. 3a.

The adenosine-H_2_ proton is sensitive to the nature of both the 5′- and 3′-nucleotides (Cromsigt et al. 2001) and exhibits a large chemical shift range of ~6.4–8.0 ppm. Importantly, the simultaneous presence of a 5′-pyrimidine and 3′-purine is associated with a significant upfield A-H_2_ NMR chemical shift, to a less crowded region of the RNA NMR spectrum (6.4–7.1 ppm, Fig. 2b) where they are potentially useful for structural characterization of large RNAs Lu et al. (2011). In contrast, significant downfield shifts are observed for the H_2_ protons of adenosines that are preceded by a purine and followed by a pyrimidine, Fig. 2b. The H_5_ protons of the C and U are sensitive to the nature of the preceding residue of the triplet but exhibit almost no detectable sensitivity to the nature of the following residue, Fig. 3c, d. The pyrimidine H_6_ protons are also more sensitive to the nature of the 5′ residue, but exhibit some sensitivity to the 3′ residue as well (Fig. 3c, e). The ribose protons appear to be sensitive to the nature of both the 5′ and 3′ residues, although the limited chemical shift dispersion and uncertainties regarding some of H_2_′ and H_3_′ assignments make it more difficult to identify clear chemical shift trends.

### Influence of 5′- and 3′-terminal base pairs within the WC-BP triplet

**Fig. 5:**
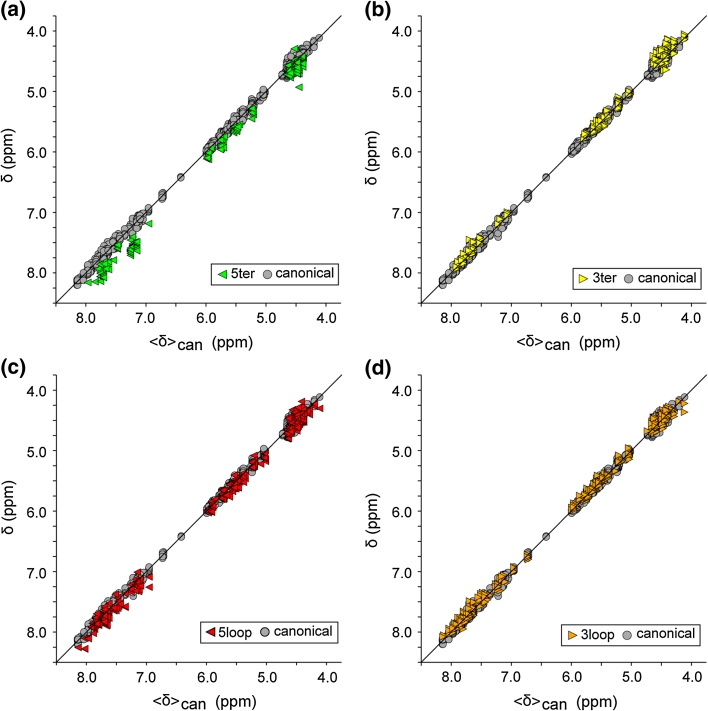
Plots of δ versus 〈δ〉_can_ (defined in Figs. 1, 2 captions) for the central residues of WC-BP triplets that contain a 5′-terminal base pair (5ter), a 3′-terminal base pair (3ter), or are preceded and/or followed by non-canonical loops or bulges (5loop and 3loop, respectively). Symbols are defined in the panel *insets*

The presence of 5′- and/or 3′-terminating base pairs within the WC-BP triplet has a significant influence on the chemical shifts of the central residue. As shown in Fig. 5a, the aromatic, H_1_′, H_2_′ and H_3_′ protons of the central residue exhibit small but significant downfield shifts relative to 〈δ〉_can_ values when adjacent to a 5′-terminating base-paired residue (the single H_3_′ outlier is most likely due to a misassignment or typo). The most significant perturbations are observed for the aromatic protons, which exhibit deviations in the range of 0.15–0.45 ppm. In contrast, most signals for residues that reside next to a 3′-terminal WC-BP exhibit smaller but nevertheless consistent upfield shifts relative to the 〈δ〉_can_ values, Fig. 5b. The most significant shifts are observed for H_2_′ protons which have a mean upfield shift of 0.2 ppm.

### Influence of non-canonical elements adjacent to the WCBP triplets

Our analysis assessed the influence of non-canonical structural elements that reside immediately upstream (5loop) or downstream (3loop) of the WC-BP triplets. We defined these elements to include internally stacked residues that are not involved in Watson–Crick base pairing, looped or bulge residues believed to be flexible or structured (e.g., K-turns), and residues involved in base-triples or long-distance RNA–RNA interactions. As shown in Fig. 5d, the presence of non-canonical RNA structures at the 3′-end of the WC-BP triplet does not appear to significantly influence any of the proton shifts associated with the central residue of the triplet. On the other hand, the presence of non-canonical structure on the 5′-side of the WC-BP triplet results in small but significant upfield shifts relative to 〈δ〉_can_ values for the aromatic and H_1_′ protons, Fig. 5c.

### Influence of G:U base pairing within the triplet

**Fig. 6:**
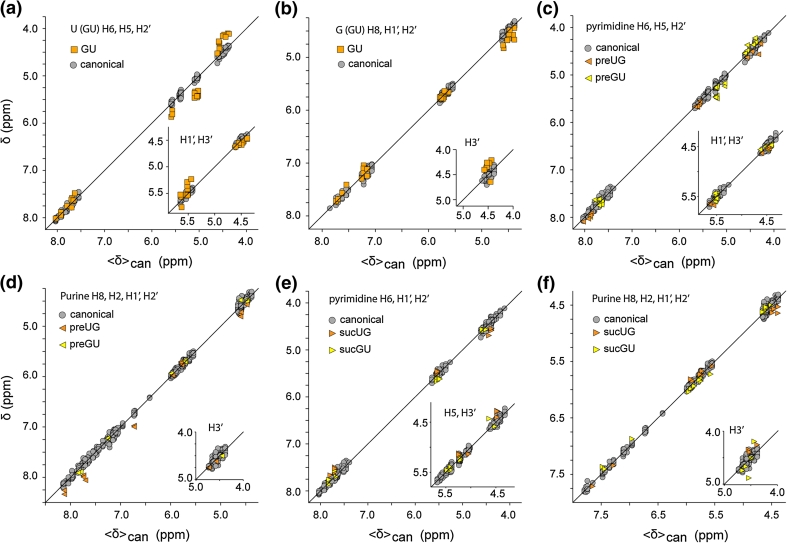
Plots showing the sensitivity of the ^1^H NMR chemical shifts to GU and UG wobble pairing within the canonical WC-BP triplet. **a** The central U of an otherwise canonical triplet is paired with G. **b** The central G of an otherwise canonical triplet is paired with U. **c–f** Influence of GU base pairs at the n_(i−1)_ and n_(i+1)_ position of the n_(i−1)_−N_i_−n_(i+1)_ triplet on the NMR chemical shift of the central canonical base pair. Symbols are defined in the panel *insets*

Because GU base pairs are both prevalent and functionally important (Varani and McClain 2000), we also assessed the influence of this class of base pairing on ^1^H NMR chemical shifts. Systematic variations are apparent for some protons of the central U of triplets when they are base paired with G. Considering only canonical triplets in which the central U:A base pair is substituted by U:G, the H_5_ protons exhibit a downfield shift and the H_1_′ and H_2_′ protons exhibit small upfield shifts, whereas the H_6_ and H_3_′ chemical shifts appear to be relatively unperturbed, Fig. 6a. If the central residue of the canonical triplet is a G, base pairing with U results in a small downfield shift of the H_2_′ NMR signal and upfield shift of H_3_′ (relative to base pairing with C) but does not significantly affect the shifts of the other G protons, Fig. 6b.

The presence of GU (or UG) base pairs at the n_(i−1)_ or n_(i+1)_ positions can significantly influence the signals of the central residue, and data for otherwise canonical triplets are shown in Fig. 6c–f. For triplets in which the central residues is a pyrimidine, the H_1_′ and H_3_′ are relatively unaffected by the presence of a preceding GU wobble, Fig. 6c. However, the H_6_, H_5_ and H_2_′ protons are systematically perturbed, with the u(wob)-U/C-n H_6_ signal shifted downfield, the g(wob)-U-n H_6_ signal shifted upfield, and the g(wob)-C-n C-H_6_ signal shifted downfield relative to the average canonical shifts, Fig. 6c. Interestingly, the u(wob)-C/U-n H_5_ shifts are relatively unperturbed relative to canonical shifts, whereas g(wob)-C/U-n H_5_ shifts are generally shifted downfield relative to the signals observed for the canonical triplets, Fig. 6c. Also, H_2_′ shifts of the central pyrimidine are shifted downfield when preceded by a UG wobble, but are shifted upfield when preceded by a GU wobble, Fig. 6c. When the central residue is a purine, the H_1_′ and H_3_′ proton shifts are relatively unaffected by a preceding wobble, but the H_8_, H_2_, and H_2_′ protons generally exhibit systematic downfield shifts, with the magnitude of the shift being somewhat greater for a preceding U(wob) compared to a preceding G(wob), Fig. 6d.

The presence of a subsequent GU wobble can also result in systematic chemical shift perturbations. For triplets in which the central residue is a pyrimidine followed by a U(wob) mismatch, the H_6_ and H_3_′ signals exhibits small upfield shifts but the remaining signals do not appear to be significantly perturbed, Fig. 6e. In contrast, the presence of a subsequent G(wob) mismatch does not appear to lead to any detectable perturbations, Fig. 6e. For triplets in which the central residue is a purine, a subsequent G(wob) leads to a small systematic downfield shift of the H_1_′ proton but does not significantly perturb the other NMR signals, whereas a subsequent U(wob) pair results in small upfield shifts of the H_6_ and H_5_ signals and a small downfield shift of the H_2_′ signal, Fig. 6f.

### Chemical shift predictions

**Fig. 7:**
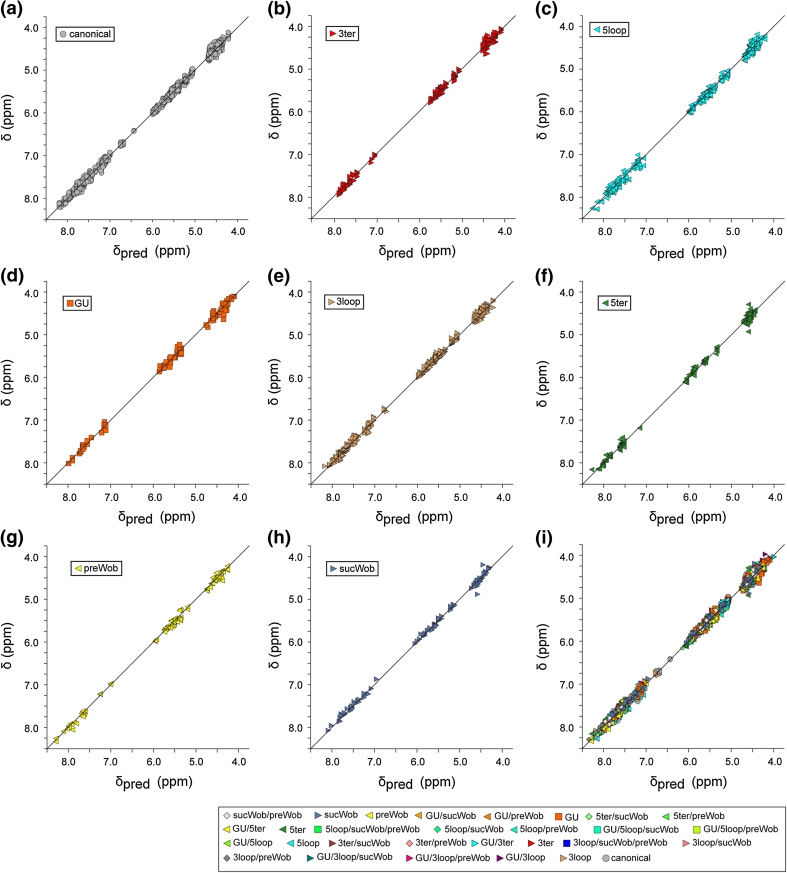
Plots of δ versus predicted chemical shift (δ_pred_), calculated by Pace regression as described in the text. **a**–**h** Data for triplets that are fully canonical (**a**) (rmsd = 0.050) or include a single non-canonical element, **b** 3′-terminal residue (3ter), rmsd = 0.054; **c** loop, bulge or stacked non-BP residue immediately 5′ to the triplet, rmsd = 0.066; **d** GU wobble at the center of an otherwise canonical triplet (GU), rmsd = 0.073; **e** loop, bulge or stacked non-BP residue immediately 3′ to the triplet (3ter), rmsd = 0.053; **f** 5′-terminal residue within the triplet (5ter), rmsd = 0.054; **g** 5′-residue of the triplet is involved in a GU wobble interaction (preWob), rmsd = 0.057; **h** 3′-residue of the triplet is involved in a GU wobble interaction (sucWob), rmsd = 0.057; **i** all data, including triplets with multiple non-canonical elements, rmsd = 0.056

The Pace regression approach described above provided predicted chemical shift values for all possible combinations of WC-BP triplet parameters used in the present study, Table 1. ^1^H NMR chemical shifts observed for the canonical triplets are in good agreement with the shifts predicted using the Pace regression approach described above (δ_pred_), Fig. 7a (rms deviation = 0.050). Excellent agreement was also observed for triplets that contained only a single modifying element (e.g., only a 5ter but no other non-canonical elements), with the greatest deviations observed for a few of the H_2_′ and H_3_′ assignments, Fig. 7b–h (rms deviation in the range 0.057–0.057). Good fits were also observed for triplets that contained more than one modifying element (rms deviation for all canonical and non-canonical triplets = 0.056), Fig. 7i. As observed in other fits, the largest deviations are observed for the H_2_′ and H_3_′ proton assignments.

The data in Table 1 can be used in computer programs such as NMRView (Johnson 2004; Johnson and Blevins 1994) or ad hoc calculations to predict chemical shifts. The constant term represents the value of the given atom in nucleotide i, when the i − 1 and i + 1 nucleotides are both U, and all nucleotides from i − 2 through i + 2 are present and in canonical Watson–Crick base pairs. For example, an A-H_2_ proton, in a canonical uAu triplet would be at 7.0299. Calculating the shift of the A-H_2_ proton in a different environment is done by adding to the constant term the contributions from any applicable columns in the A-H_2_ row of Table 1. For example, the chemical shift of an A-H_2_ proton in a gAc triplet, in which the i − 2 residue is in a loop, would be: 7.8469 ppm (7.0299 + 0.6899 + 0.0658 + 0.0622). If the i − 1 G is in a GU (rather than GC) base pair, the A-H_2_ proton chemical shift would be: 7.9217 ppm (7.0299 + 0.7637 + 0.0658 + 0.0622).

## Conclusions

The present studies provide the first quantitative analysis of the RNA non-exchangeable ^1^H NMR chemical shifts in the BMRB. Our studies identify sequence-dependent chemical shift correlations and establish the influence of terminating base pairs within the triplets and canonical and non-canonical structures adjacent to the BP triplets (i.e. bulges, loops, WC and non-WC BPs). Excellent correlations were observed despite the fact that the NMR data were obtained under different conditions of pH, buffer, ionic strength, and temperature. A relatively small number of outliers that were not utilized in the analysis, mainly ribose H_2_′ and H_3_′ assignments, are likely due to assignment or typographical errors and should be re-examined. Assignments for some triplet combinations were either limited or lacking; for example, the database does not include assignments for two of the 64 possible “canonical triplets.” Although shifts for these triplets could be predicted from assignments made for non-canonical triplets (e.g., WC-BP triplets adjacent to non-canonical structures or that contain terminal or GC base pairs), future studies of oligonucleotides with the missing sequences are clearly in order.

The statistics indicate that the protocol employed for chemical shift predictions, assigning attributes to different triplet environments and then conducting selection and linear model fitting with Pace Regression, performed very well for the data used in this study. However, as we move forward with this research and the number of attributes is expanded, alternative fitting methods such as Neural Networks and allowing attributes to contribute in non-linear modes may be required. The protocol used here can, of course, also be applied to nitrogen and carbon nuclei, and it will be interesting to determine if these nuclei exhibit similar environment- and structure-dependent sensitivities.

The ^1^H NMR shifts observed for residues that participate directly in long-range RNA–RNA interactions or interactions with ligands or proteins, as identified in the associated publications and/or the structure coordinate (PDB) files, generally deviated from the A-form helical triplet shifts. For example, the H_6_ and H_5_ NMR chemical shifts observed for residue U5 of the ScYLV P-1-P2 frameshifting pseudoknot (7.93 and 5.25 ppm, respectively) (Cornish et al. 2005), deviate by 0.24 and 0.29 ppm from the expected values (7.69 and 5.54 ppm, respectively) and are well outside the rms range calculated for canonical gUg triplets (rms = 0.06 and 0.03 ppm, respectively). Significant deviations were also observed for otherwise canonical A-form helical residues that interact with protein elements. In future studies of RNAs with unknown structures, the observation of outlier chemical shifts may serve as useful indicators of potential long-range RNA:RNA or RNA:protein interactions. In addition, the trends identified in the present studies should facilitate the refinement of algorithms used to calculate ^1^H NMR chemical shifts on the basis of RNA structural coordinates alone (Cromsigt et al. 2001; Case 1995, 2002; Dejaegere et al. 1999; Case et al. 2005), thereby making the ^1^H NMR chemical shift a more useful parameter for RNA structure refinement. Because the variations in chemical shifts observed for atoms of a given triplet are small, variations in the 3D structures of the triplets should also be small. This observation lends support for refinement approaches that utilize residual dipolar couplings and/or small angle X-ray scattering (SAXS) data to orient idealized A-form helices (Funari et al. 2000; Walsh et al. 2004; Zuo et al. 2008; Grishaev et al. 2008; Wang et al. 2009, 2010; Burke et al. 2012).

In the course of these studies, chemical shift trends were tentatively identified for a number of non-A-form helical structures that are well represented in the BMRB, particularly those of conserved tetraloops (e.g., GNRA). Future studies that include parameterizations for tetraloops, base triples, and other conserved and well-defined RNA sub-structures will likely lead to the identification of additional trends useful for ^1^H NMR assignment and verification. In addition, it should now be possible to incorporate the approach into software programs to enable semi-automated assignment of RNA, including large RNAs with different combinations of ^2^H-labeled or segmentally-labeled nucleotides (underway).

## Electronic supplementary material

Below is the link to the electronic supplementary material.

Supplementary material 1 (DOC 55 kb)

## Abbreviations

BMRB: Biological Magnetic Resonance Data Bank
NDB: Nucleic Acid Database
PDB: Protein Data Bank
WC-BP: Watson–Crick base pair
G/g: Guanosine
N/n: Any nucleotide
R/r: Purine
Py/py: Pyrimidine
A/a: Adenosine
C/c: Cytosine
U/u: Uridine
〈δ〉: Mean NMR chemical shift
〈δ〉_can_: Mean NMR chemical shift determined for a canonical triplet, defined here as a stretch of three sequential canonical base pairs that is both preceded and followed by at least one canonical base pair
δ_pred_: Predicted NMR chemical shift
