# Supplementary material for: Database proton NMR chemical shifts for RNA signal assignment and validation
Source: J Biomol NMR. 2012 Nov 23;55(1):33–46. doi: 10.1007/s10858-012-9683-9 (PMC3555346; doi:10.1007/s10858-012-9683-9)
Supplement: Supplementary file 1 — Supplementary material 1 (DOC 55 kb) [file 10858_2012_9683_MOESM1_ESM.doc]

**SUPPLEMENTARY MATERIAL**

**Database Proton NMR Chemical Shifts for RNA Signal Assignment and Validation**

Shawn Barton^1,2^, Xiao Heng^1,2^, Bruce Johnson^2,3,*^ and Michael F. Summers^1,2,*^

^1^Howard Hughes Medical Institute and ^2^Department of Chemistry and Biochemistry, University of Maryland Baltimore County, 1000 Hilltop Circle, Baltimore, MD 21250, and ^3^One Moon Scientific, Inc., 839 Grant Ave., Westfield, NJ 07090

^*^Corresponding authors: [johnsonb@umbc.edu](mailto:johnsonb@umbc.edu); [summers@hhmi.umbc.edu](mailto:summers@hhmi.umbc.edu)

**Table S1. Representative input file used by RNAShifts**

# FIRST LINE = BMRB ID# #Sample_Conditions (blank if one)

# SECOND LINE = residue 1 (5´-terminus); followed by sequential residue numbers

# LAST LINE = 3´-terminus

# RESIDUE ALONE = Participates in Watson Crick Base Pair

# OTHER INDICATORS:

#[Nucleotide] [base paired res] [structure] [other influence: opp strand neighbor, protein, ligand]

# [G,C,A,U,X] [G,C,A,U,X,-] [wc, mismatch, loop, stacked, NNNN-#, 3prime-nc, 5prime-nc, kissing,

# A-minor, triple, GC-U-triple, AA-U-triple] [

#

# Definitions:

# gaga = GNRA tetraloop

# guga = GNRA tetraloop

# guaa = GNRA tetraloop

# gaaa = GNRA tetraloop

# guua = GNRA tetraloop

# loop = unstructured OR complex loop structure (with base triples, etc)

# 3prime-nc = 3´ neighboring basepair displaced by bulge on opposite strand

# 5prime-nc = 5´ neighboring basepair displaced by bulge on opposite strand

# X = modified or protonated base (consistent with BMRB nomenclature)

# Multiple Assignments in a single File:

# BMRB 6076 2 (TWO SAMPLE CONDITIONS: 283K and 298K)

# BMRB 6076 2-1

# BMRB 6076 2-2

# ACTUAL SAMPLE INPUT:

BMRB 6239

G

G

U G

G A

A C

C

G

C

C

G - GUAA-1

U - GUAA-2

A - GUAA-3

A - GUAA-4

G

G

C

G

C A

A G

G U

C

C

BMRB 15342

C

C

U

C

C

C - loop

U - loop

X - loop

A - loop

C - loop

A - loop

A - loop

G

G

A

G

G

**Table S2. BMRB NMR data that were either re-assigned or excluded from analysis^a^**

**BMRB No. of Lists List Residue Assignment δ (or blank to drop)**

**Modified assignments:**

0004 1 1 3 H_1_´ 5.571

0004 1 1 7 H_3_´ 4.494

0004 1 1 7 H₂´ 4.596

4745 1 2 9 H_1_´ 5.82

4745 2 2 10 H_1_´ 5.76

4745 2 2 11 H₅ 5.18

4745 2 2 11 H_1_´ 5.46

6094 1 1 21 H₅ 5.415

6094 1 1 27 H₅ 5.701

6094 1 1 27 H_1_´ 5.670

6094 1 1 83 H₅ 5.435

15319 1 1 2 H₆ 7.976

15362 1 1 3 H₅ 5.45

15331 1 1 3 H₅ 5.45

17083 1 1 28 H₅ 5.610

17083 1 1 28 H_1_´ 5.658

17083 1 1 62 H_1_´ 5.419

17682 1 1 11 H₈ 7.27

17682 1 1 30 H_1_´ 5.55

**Misassignments or typos that were removed manually:**

15331 1 1 2 H_3_´

15113 1 1 14 H₂´

15113 1 1 69 H₈

15113 1 1 69 H_1_´

15113 1 1 70 H_1_´

15113 1 1 72 H_1_´

17083 1 1 3 H_3_´

17083 1 1 28 H₆

**Outliers that were dropped automatically by RNAShifts:**

6485 1 1 5 H₂

10014 1 1 2 H₈

4867 2 2 20 H₈

5007 1 1 20 H₅

6633 1 1 12 H₅

17188 1 1 14 H₅

17520 1 1 14 H₅

4750 1 1 25 H₅

5919 1 1 13 H₆

15417 1 1 25 H₆

6652 1 1 25 H₆

4120 1 1 14 H₆

16479 1 1 28 H_1_´

17682 1 1 2 H_1_´

4867 2 2 20 H_1_´

5007 1 1 20 H_1_´

16479 1 1 29 H_1_´

6115 1 1 33 H_1_´

15856 1 1 22 H_1_´

4867 2 2 21 H_1_´

7090 1 1 17 H_1_´

17682 1 1 2 H₂´

17682 1 1 11 H₂´

5007 1 1 20 H₂´

5007 1 1 29 H₂´

6115 1 1 33 H₂´

4867 2 2 21 H₂´

7090 1 1 17 H₂´

11014 2 2 3 H₂´

5703 1 1 2 H_3_´

5962 1 1 13 H_3_´

4120 1 1 3 H_3_´

7090 1 1 16 H_3_´

6115 1 1 4 H_3_´

4745 1 2 10 H_3_´

4745 2 2 11 H_3_´

15538 1 1 28 H_3_´

15858 2 2 6 H_3_´

**^a^**Assignments from BMRB files that were either modified (with new assignment shown in last column) or dropped (no now assignments shown). Modifications to correct for typographical errors and possible misassignments were based on assessments of spectra in relevant publications. Columns from left to right contain the BMRB ID number; the number of assignment tables in the BMRB file (due to different sample conditions, sequences, etc.); the specific assignment list in the BMRB file; the residue number; the proton assignment; modified assignment (if modified; blank if the assignment was dropped).

**Table S3. Referencing corrections determined by Pace Regression analysis**

**BMRB^a^ Mean^b^ Std. Dev.^c^ Median^d^ Sample Size^e^**

0001.1.1 0.004 0.045 0.019 19

0002.1.1 -0.000 0.050 0.002 35

0003.1.1 0.000 0.059 -0.003 49

0004.1.1 0.005 0.064 0.013 39

4120.1.1 -0.016 0.064 -0.013 16

4125.1.1 -0.011 0.038 -0.005 34

4135.1.2 -0.010 0.063 -0.003 18

4135.2.2 0.002 0.040 0.010 20

4175.1.1 0.005 0.046 -0.002 57

4226.1.1 -0.001 0.048 -0.003 56

4250.1.1 0.003 0.041 0.006 47

4345.1.1 -0.000 0.096 -0.013 29

4346.1.1 -0.005 0.086 0.001 19

4745.1.2 0.001 0.068 -0.003 36

4745.2.2 -0.003 0.050 0.001 17

4750.1.1 0.004 0.099 -0.032 26

4780.1.1 -0.002 0.045 -0.011 7

4867.2.2 -0.018 0.130 0.017 14

5007.1.1 0.021 0.083 0.006 36

5256.1.1 -0.003 0.040 0.000 27

5259.1.1 -0.003 0.046 -0.005 27

5321.1.1 -0.003 0.062 0.001 55

5371.1.1 0.001 0.039 0.004 19

5528.1.1 -0.001 0.046 -0.004 13

5531.1.1 -0.002 0.036 -0.004 18

5553.1.1 -0.013 0.073 0.010 10

5559.1.1 -0.005 0.053 0.005 33

5632.1.1 -0.008 0.072 0.000 29

5655.1.1 0.005 0.054 0.000 28

5703.1.1 0.001 0.032 -0.002 18

5705.1.1 0.002 0.042 0.003 28

5773.1.1 -0.003 0.054 -0.005 94

5834.1.1 -0.004 0.057 -0.005 63

5852.1.1 -0.009 0.059 -0.006 19

5919.1.1 -0.001 0.048 -0.007 72

5932.1.1 -0.012 0.038 -0.015 28

5962.1.1 0.000 0.051 0.005 77

6062.1.1 -0.011 0.049 -0.022 37

6076.1.1 -0.002 0.061 0.002 56

6077.1.1 -0.002 0.057 0.002 55

6094.1.1 -0.001 0.056 0.003 127

6115.1.1 -0.008 0.095 -0.003 40

6239.1.1 0.001 0.050 0.003 18

6320.1.1 -0.010 0.052 -0.017 29

6485.1.1 0.006 0.055 -0.004 81

6543.1.1 -0.001 0.055 -0.001 109

6562.1.1 0.002 0.047 -0.002 47

6633.1.1 -0.004 0.056 -0.005 102

6652.1.1 -0.002 0.075 -0.013 69

6756.1.1 -0.008 0.045 -0.012 47

7090.1.1 -0.022 0.081 -0.026 35

7098.1.1 -0.008 0.044 -0.008 52

7403.1.1 0.005 0.044 -0.001 54

7404.1.1 0.005 0.042 0.001 53

7405.1.1 0.008 0.053 0.007 37

10014.1.1 -0.038 0.074 -0.065 16

10018.1.1 -0.002 0.068 -0.013 62

11014.1.2 -0.008 0.045 -0.003 19

11014.2.2 -0.013 0.064 0.009 18

15113.1.1 -0.012 0.058 -0.006 45

15319.1.1 -0.001 0.067 -0.011 28

15331.1.1 0.000 0.055 -0.006 27

15342.1.1 -0.000 0.059 -0.007 28

15362.1.1 -0.001 0.047 -0.001 28

15417.1.1 0.004 0.043 0.014 75

15538.1.1 0.003 0.065 -0.005 48

15571.1.2 -0.003 0.049 -0.004 37

15571.2.2 -0.000 0.052 0.004 38

15572.1.2 -0.004 0.060 -0.018 28

15572.2.2 -0.007 0.071 -0.013 9

15656.1.1 0.002 0.046 0.001 10

15745.1.1 -0.004 0.048 -0.001 19

15856.1.1 0.018 0.039 0.019 66

15857.1.2 0.017 0.043 0.019 57

15858.1.2 0.021 0.065 0.023 24

15858.2.2 0.022 0.055 0.028 20

15859.1.1 0.018 0.044 0.015 66

15869.1.1 -0.006 0.053 -0.008 45

16431.1.1 0.001 0.049 0.003 18

16479.1.1 0.000 0.047 0.003 63

16654.1.1 -0.003 0.041 -0.014 18

16655.1.1 -0.003 0.040 -0.015 18

16877.1.1 -0.003 0.041 -0.003 21

16941.1.1 -0.002 0.049 -0.009 16

16951.1.1 -0.003 0.059 -0.012 9

16952.1.1 -0.003 0.077 0.035 9

16980.1.1 0.007 0.063 0.004 45

17083.1.1 -0.000 0.049 -0.002 36

17188.1.1 -0.003 0.049 0.000 65

17292.1.1 -0.017 0.064 -0.008 29

17309.1.1 0.017 0.060 0.009 20

17316.1.1 0.001 0.062 -0.001 73

17401.1.1 -0.004 0.038 -0.004 18

17436.1.1 0.002 0.044 0.005 44

17449.1.1 0.002 0.058 -0.013 28

17517.1.1 -0.004 0.045 0.003 12

17520.1.1 -0.003 0.038 -0.002 20

17572.1.1 -0.003 0.052 -0.003 21

17573.1.1 -0.006 0.097 -0.015 13

17682.1.1 -0.002 0.073 -0.010 53

17901.1.1 0.002 0.071 0.018 16

**^a^**BMRB file denoted with decimal points as follows: BMRB ID number.assignment list number.total lists per BMRB file. **^b^**Mean chemical shift reference adjustment used for subsequent data analyses. **^c^**Standard deviation calculated for chemical shift re-referencing. **^d^**Median chemical shift offset calculated during re-referencing. **^e^**Total number of data points (assignments) used to re-reference the individual BMRB dataset.
